# Megalin, Proton Pump Inhibitors and the Renin–Angiotensin System in Healthy and Pre-Eclamptic Placentas

**DOI:** 10.3390/ijms22147407

**Published:** 2021-07-10

**Authors:** Yuan Sun, Lunbo Tan, Rugina I. Neuman, Michelle Broekhuizen, Sam Schoenmakers, Xifeng Lu, A. H. Jan Danser

**Affiliations:** 1 Division of Pharmacology and Vascular Medicine, Department of Internal Medicine, Erasmus MC, 3015 CN Rotterdam, The Netherlands; sunyuan@sztu.edu.cn (Y.S.); l.tan@erasmusmc.nl (L.T.); r.neuman@erasmusmc.nl (R.I.N.); m.broekhuizen@erasmusmc.nl (M.B.); 2 Department of Pharmacology, College of Pharmacy, Shenzhen Technology University, Shenzhen 518118, China; 3 Health Science Center, Department of Physiology, Shenzhen University, Shenzhen 518061, China; x.lu@szu.edu.cn; 4 Division of Neonatology, Department of Pediatrics, Erasmus MC, 3015 CN Rotterdam, The Netherlands; 5 Department of Obstetrics and Gynaecology, Erasmus MC, 3015 CN Rotterdam, The Netherlands; s.schoenmakers@erasmusmc.nl

**Keywords:** pre-eclampsia, renin–angiotensin system, megalin, proton pump inhibitors

## Abstract

Soluble Fms-like tyrosine kinase-1 (sFlt-1) is increased in pre-eclampsia. The proton pump inhibitor (PPI) lowers sFlt-1, while angiotensin increases it. To investigate whether PPIs lower sFlt-1 by suppressing placental renin–angiotensin system (RAS) activity, we studied gene expression and protein abundance of RAS components, including megalin, a novel endocytic receptor for prorenin and renin, in placental tissue obtained from healthy pregnant women and women with early-onset pre-eclampsia. *Renin, ACE, ACE2*, and the angiotensin receptors were expressed at identical levels in healthy and pre-eclamptic placentas, while both the (pro)renin receptor and megalin were increased in the latter. Placental prorenin levels were upregulated in pre-eclamptic pregnancies. Angiotensinogen protein, but not mRNA, was detectable in placental tissue, implying that it originates from maternal blood. Ex vivo placental perfusion revealed a complete washout of angiotensinogen, while prorenin release remained constant. The PPI esomeprazole dose-dependently reduced megalin/(pro)renin receptor-mediated renin uptake in Brown Norway yolk sac epithelial cells and decreased sFlt-1 secretion from placental villous explants. Megalin inhibition blocked angiotensinogen uptake in epithelial cells. In conclusion, our data suggest that placental RAS activity depends on angiotensinogen taken up from the maternal systemic circulation. PPIs might interfere with placental (pro)renin-AGT uptake/transport, thereby reducing angiotensin formation as well as angiotensin-induced sFlt-1 synthesis.

## 1. Introduction

Pre-eclampsia is a hypertensive disorder characterized by new-onset hypertension and proteinuria occurring after 20 weeks of gestation. It is one of the major causes of worldwide maternal mortality and morbidity [1,2,3]. A normal pregnancy requires a ≈30% increase in extracellular fluid volume to provide an adequate blood supply for the developing uterus, placenta and fetus [4]. The renin–angiotensin system (RAS) plays a key role in this process. Estrogen stimulates hepatic angiotensinogen (AGT) expression, resulting in a 3–5-fold rise in circulating AGT [4,5]. The plasma renin concentration also rises modestly [6], and together with the rise in AGT, this results in a significant rise in plasma renin activity (PRA); thus, ensuring sufficient RAS activity to allow water and salt retention [7,8]. Remarkably, plasma prorenin levels increase much more strongly than plasma renin levels [6,9], due to prorenin release from the ovaries and, to a lesser degree, the placenta [10]. The function of this prorenin remains unclear.

In pre-eclampsia, the rise in renin and AGT in the circulation is suppressed, although sensitivity to angiotensin (Ang) II is enhanced [11]. Circulating prorenin levels in pre-eclamptic women are in the normal pregnancy range [12]. Given the local production of prorenin in the placenta, while the placenta is a key player in the development of pre-eclampsia, disturbed placental RAS activity may contribute to this phenomenon. Yet, data on prorenin levels in the uteroplacental unit of pre-eclamptic women are conflicting, with evidence for decreases, increases and no alteration [13,14,15]. What exactly determines the distribution of prorenin across the ovaries and uteroplacental unit, as well as its release into the blood stream, is unknown. It may require transcellular transport. For instance, amniotic fluid prorenin is of chorionic origin [16], implying that chorionic prorenin is capable of crossing the amnion membrane. A novel player in this field is megalin. Megalin, a recently discovered endocytic receptor for prorenin and renin, not only contributes to renin/prorenin reabsorption in the proximal tubule of the kidney, but simultaneously plays a role in renal Ang II generation, although how exactly this occurs is still unknown [17]. Outside the kidney, megalin is present in syncytiotrophoblast and cytotrophoblast cells in the placenta [18]. Membrane recycling of megalin is a fast process which relies on vacuolar H^+^-ATPase (V-ATPase)-dependent endosomal acidification [19]. The latter involves the so-called (pro)renin receptor ((P)RR), which is an accessory protein of V-ATPase. Here, it is of interest to note that proton pump inhibitors (PPIs) have been observed to lower the placental release of soluble Fms-like tyrosine kinase-1 (sFlt-1), a mediator of maternal endothelial dysfunction in pre-eclampsia [20,21]. Ang II is a stimulator of sFlt-1 [22]. Although PPIs are capable of inhibiting V-ATPase [23,24], such inhibition did not contribute to their acute effects on sFlt-1 release from human trophoblast cells [25]. A further possibility is that PPIs affect sFlt-1 production indirectly, by preventing RAS component transport/uptake.

In the present study, we, therefore, set out to first quantify the expression of major RAS components (including megalin and (P)RR) and their protein abundance in healthy and pre-eclamptic placentas. We focused on early-onset pre-eclampsia (EoPE), and not late-onset pre-eclampsia, since the latter has a different pathophysiological mechanism, being more a maternal rather than a placental syndrome, and showing clear histopathological differences.

Next, we studied the release of RAS components from ex vivo-perfused placentas of healthy and pre-eclamptic pregnancies. Third, we verified whether PPIs modulate megalin-dependent renin uptake in megalin-expressing Brown Norway yolk sac epithelial cells (BN16 cells). Finally, we investigated whether PPIs regulate sFlt-1 secretion in both healthy and pre-eclamptic placental explants.

## 2. Results

Clinical characteristics of the patients are provided in Table 1. The data confirm that our PE patients fulfilled the ISSHP 2018 criteria [26].

### 2.1. Expression of RAS Components in Healthy and Pre-Eclamptic Placentas

**Figure 1:**
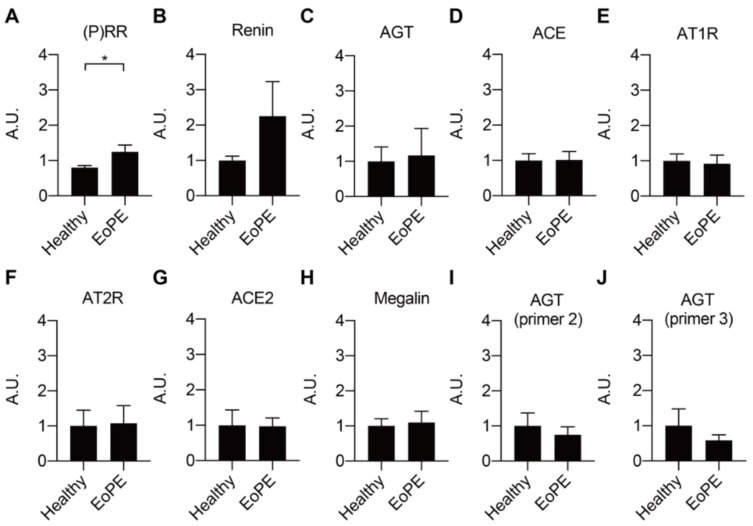
Gene expression levels of RAS components (**A**–**G**) and megalin (**H**) in healthy and early-onset pre-eclamptic (EoPE) placental tissue. (**I**,**J**): *AGT* expression was confirmed with different pairs of primers. Data are mean ± SEM of *n* = 12. * *p* < 0.05. (*P)RR*, (pro)renin receptor; *AGT*, angiotensinogen; *ACE*, angiotensin-converting enzyme; *AT1R*, angiotensin II type 1 receptor; *AT2R*, angiotensin II type 2 receptor; A.U., arbitrary unit.

**Figure 2:**
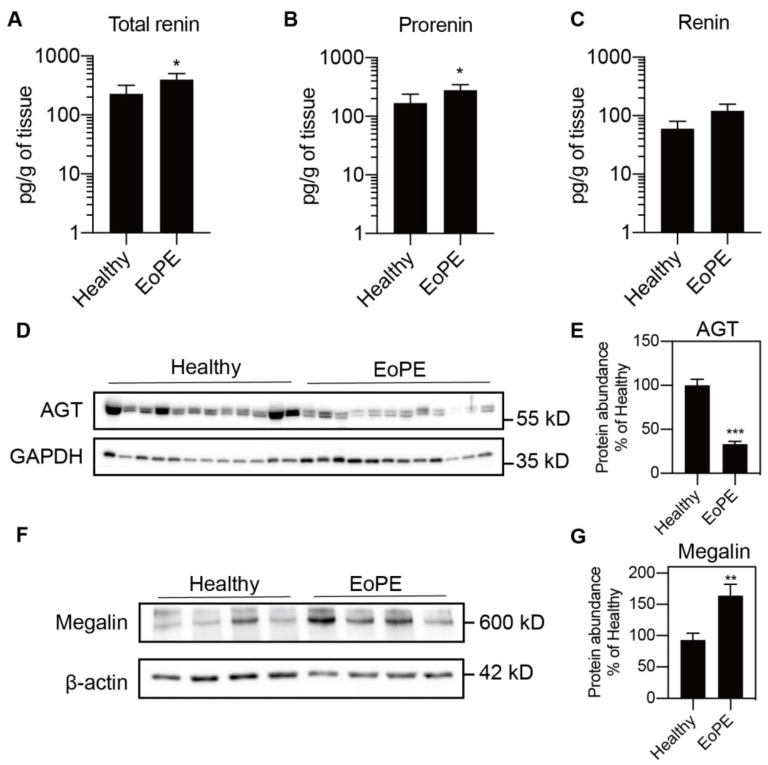
Total renin (**A**), renin (**B**) and prorenin (**C**) levels (*n* = 16) measured by immunoradiometric assay and angiotensinogen (AGT; **D**,**E**) and megalin (**F**,**G**) protein abundance (*n* = 12) measured by Western blot in healthy and early-onset pre-eclamptic (EoPE) placental tissue. Panels (**D**,**F**) are representative blots of AGT and megalin protein. Data were normalized versus GAPDH (**E**) or β-actin (**G**). Data are mean ± SEM. * *p* < 0.05, ** *p* < 0.005, *** *p* < 0.0005 vs. Healthy. AGT, angiotensinogen.

Placental expression of *renin*, *AGT*, angiotensin-converting enzyme (*ACE*), *ACE2*, the Ang II type 1 and type 2 receptor (*AT1R*, *AT2R*), and *megalin* was comparable between healthy and pre-eclamptic placentas (*n* = 12 for each), while (P)RR expression was increased in the latter (Figure 1A–H). *AGT* expression was at or below the detection limit (Ct = 38) in most of the samples, even when using two additional sets of primers (Figure 1I,J). Total renin levels in healthy placental tissue amounted to 118 (range 42.5–1476) pg/g tissue, and 72 ± 2% of this was prorenin (Figure 2A–C; *n* = 16). Total renin levels were significantly (*p* < 0.05) higher in pre-eclamptic placentas (266 (range 54.5–1568) pg/g tissue), and this was due to upregulated prorenin (*p* < 0.05) rather than renin. Nevertheless, the proportion of renin in the pre-eclamptic group (29 ± 2%) was not different from that in healthy placentas. Placental AGT abundance was decreased (*p* < 0.0001) in pre-eclamptic placentas (Figure 2D,E), while the opposite was true (*p* < 0.01) for megalin protein abundance (Figure 2F,G).

### 2.2. Placental Release of (Pro)renin and AGT

**Figure 3:**
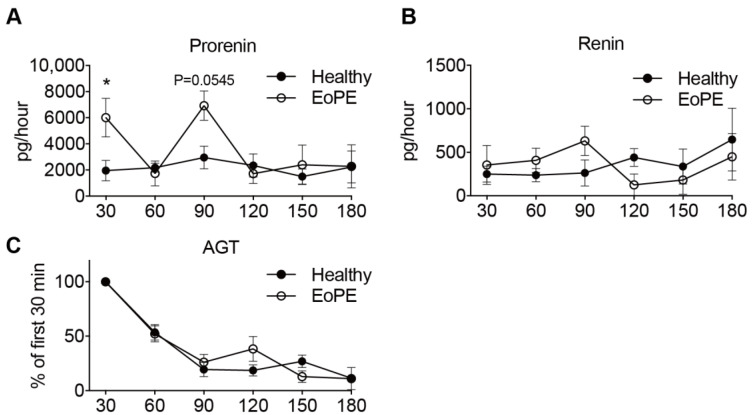
Prorenin (**A**), renin (**B**) and angiotensinogen (**C**) release from isolated perfused healthy (*n* = 5) and early-onset pre-eclamptic (EoPE, *n* = 6) cotyledons. AGT: angiotensinogen. Perfusate collection from two of the pre-eclamptic placentas could not be continued over the full 180 min due to fetal-to-maternal leakage. In such cases, only the samples prior to the occurrence of leakage were used. Data are mean ± SEM. * *p* < 0.05.

Prorenin release into the maternal perfusate from both healthy (*n* = 5) and pre-eclamptic (*n* = 6) placentas remained stable over the entire perfusion period. In two pre-eclamptic placenta perfusions, the perfusion had to be stopped after 90 min because of leakage. Release of prorenin tended to be higher from pre-eclamptic placentas, although significance was reached at two time points only (Figure 3A). Renin release paralleled prorenin release, albeit at >10-fold lower levels (Figure 3B). In contrast, placental AGT release from both healthy and pre-eclamptic placentas peaked initially, and then displayed a steady decline, so that the release was lowest after 180 min (Figure 3C). Neither for renin, nor for AGT, were there differences observed between healthy and pre-eclamptic placentas.

### 2.3. Megalin Internalizes AGT and PPI Decreases Renin Internalization but Not Binding in BN16 Cells

**Figure 4:**
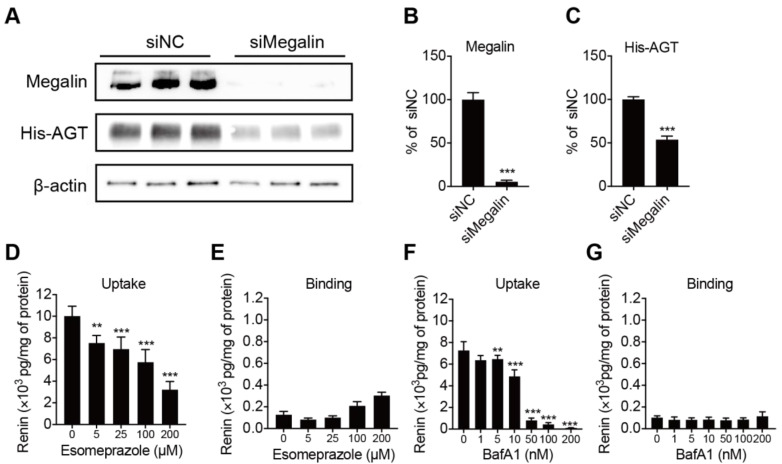
Panels (**A**–**C**): representative blots of His-AGT protein abundance in BN16 cells treated with negative control (siNC) or siRNA against megalin (siMegalin). Panels, (**D**–**G**): cell-associated renin levels after incubating BN16 cells with 100 U/L renin at 37 °C (**D**,**F**) or 4 °C (**E**,**G**) for 2 h in the presence of increasing concentrations of esomeprazole (**D**,**E**) or bafilomycin A1 (**F**,**G**). Data are mean ± SEM of *n* = 5–9. ** *p* < 0.005, *** *p* < 0.0005 vs. control.

Megalin is known to be a receptor for both renin and prorenin [27]. To verify whether it also binds and internalizes AGT, we incubated BN16 cells with tagged AGT. BN16 cells accumulated AGT following a 2 h incubation period (Figure 4A). Inhibiting megalin expression greatly suppressed this accumulation, confirming that megalin also underlies AGT uptake (Figure 4B,C). We next tested whether the PPI esomeprazole regulates renin binding and internalization. Esomeprazole dose-dependently reduced renin uptake in BN16 cells, but did not affect renin binding (Figure 4D,E). This fully paralleled the effect of bafilomycin A1 (Figure 4F,G), and illustrates that esomeprazole, such as bafilomycin A1, interferes with the megalin-mediated internalization process.

### 2.4. PPI Reduces sFlt-1 Secretion in Human Placental Villous Explants

**Figure 5:**
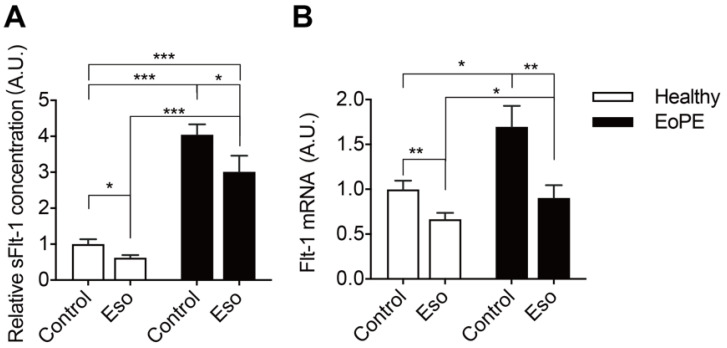
sFlt-1 levels in medium (**A**) and mRNA levels in explants (**B**) after incubating healthy or pre-eclamptic (EoPE) placental villous explants with 100 μM of esomeprazole (Eso) for 48 h. sFlt-1 level: healthy *n* = 18, EoPE *n* = 8; Flt-1 mRNA level: healthy *n* = 7, EoPE *n* = 4. * *p* < 0.05, ** *p* < 0.005, *** *p* < 0.0005 vs. 0. A.U., arbitrary unit.

As expected, pre-eclamptic placenta explants release more sFlt-1 than healthy placenta explants (Figure 5A). This is in agreement with the increased Flt-1 expression in pre-eclamptic explants (Figure 5B). Esomeprazole-suppressed Flt-1 expression and sFlt-1 release particularly in pre-eclamptic explants and, as a consequence, Flt-1 expression in pre-eclamptic explants after this drug was identical to that in healthy placenta explants.

## 3. Discussion

The existence of a placental RAS has been proposed decades ago [28]. Although it is believed that it may contribute to pre-eclampsia, its exact role is still unknown. This relates to the fact that we still do not fully understand how local Ang II synthesis occurs in the placenta. In the current study, we found that the mRNA levels of RAS components are comparable in healthy and pre-eclamptic placentas. This agrees with previous studies showing that *renin*, *AGT*, *ACE* and *AT2R* expression are similar in normal and pre-eclamptic decidua basalis [15,29,30]. Simultaneously, uteroplacental AT1R [30,31,32] and renin [32] upregulation have also been claimed to be involved in the pathogenesis of pre-eclampsia. One explanation for these conflicting observations is that different tissues were studied, ranging from endometrium and myometrium to decidual tissue. In our study, we collected tissue from the maternal side of the placenta, i.e., decidua basalis and part of chorionic villi. A second explanation is that placental RAS expression may change with increasing gestational age [32]. Thus, the 10-week difference in gestational age between the healthy and pre-eclamptic women of the current study is a confounding factor. Nevertheless, it is important to note that symptoms in early-onset pre-eclampsia (before 34 ± 0 weeks of gestation, such as in our study), develop much earlier [33], implying that changes in placental RAS expression, if having a causal effect, may be more drastic at this early gestational age.

Importantly, placental *AGT* expression in the present study was low and in most cases undetectable. Such low expression was observed earlier, when making a comparison with prolactin and renin using Northern blot analysis [34]. In situ mRNA detection via the padlock probes method also did not detect *AGT* expression in the placenta [35]. Despite the absence of significant expression, AGT protein was easily detectable in placental tissue. This implies that placental AGT must have been taken up from maternal blood. In agreement with this concept, placental AGT levels were lower in pre-eclamptic women, who are known to have greatly diminished circulating AGT levels. Moreover, perfusing the placenta with buffer resulted in a washout of AGT. Animal data similarly support a major role for AGT of maternal origin. When female mice carrying the human renin transgene were crossed with male mice carrying the human AGT transgene (both of which do not react with their mouse counterparts), the pregnant mice remained normotensive. Yet, when crossing female human AGT transgenic mice with male human renin transgenic mice, the pregnant mice did develop a pre-eclamptic phenotype, characterized by hypertension, elevated sFlt-1 levels and proteinuria [36]. Moreover, specifically inhibiting human AGT expression in the maternal liver with liver-targeted siRNA in the latter model suppressed the pre-eclamptic phenotype [37]. Taken together, these data imply that placental angiotensin generation relies on maternal AGT.

Unlike AGT release, which peaked immediately and then decreased, placental (pro)renin release remained stable over a 3 h perfusion period in healthy placentas. Since the total renin release in healthy placentas amounted to ≈2000 pg/h, and considering that a cotyledon wet weight amounted to ≈25 g, the total renin amount released during the 3 h perfusion period can be estimated to be 2500/25 × 3 = 300 pg/g. This is 2–3 times the amount detected in placental tissue. These data, therefore, strongly support the continuous synthesis and release of (pro)renin from the placenta, in full agreement with previous findings [28,38,39]. Importantly, the circulating renin levels in pre-eclamptic women are lower than those in healthy pregnant women, while their circulating prorenin levels mimic those in healthy pregnancy [12]. The present study observed 2-fold higher prorenin levels in pre-eclamptic placentas, while placental renin was identical in healthy and pre-eclamptic women. Placental (pro)renin release paralleled these latter observations. Taken together, these data suggest that placental (pro)renin synthesis occurs independently of renal (pro)renin synthesis. Yet, whether this results in independent upregulation of Ang II generation at placental tissue sites cannot be concluded, particularly since this would rely on the uptake of circulating AGT, which is lower in pre-eclampsia. Here, there might be a role for the megalin/V-ATPase pathway.

**Figure 6:**
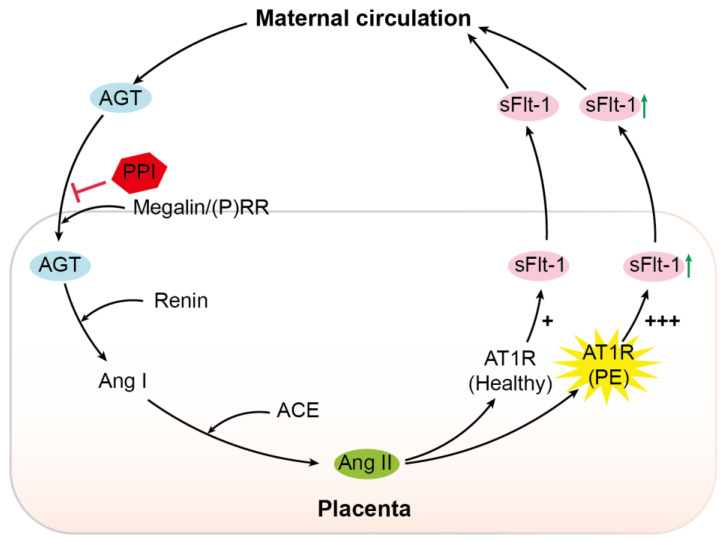
Schematic overview. AGT, angiotensinogen; 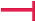, inhibition; PPI, proton pump inhibitor; (P)RR, (pro)renin receptor; Ang I, angiotensin I; ACE, angiotensin-converting enzyme; Ang II, angiotensin II; AT1R, angiotensin II type 1 receptor; PE, pre-eclampsia; sFlt-1, soluble Fms-like tyrosine kinase-1; +, normal sensitivity to Ang II; +++, increased sensitivity to Ang II.

The (P)RR is an accessory protein of the V-ATPase. Its upregulation in pre-eclampsia, combined with increased megalin levels, favors increased activity of the megalin–V-ATPase pathway in this condition [17,40]. We recently reported that megalin is a novel endocytic receptor for (pro)renin in the kidney [27]. Inhibition of megalin and the (P)RR similarly suppressed endocytosis, without showing additive effects [25]. This confirms that megalin-mediated uptake relies on (P)RR/V-ATPase-dependent endosomal acidification. These data were obtained in BN16 cells, which predominantly express megalin, and no other renin/prorenin-binding receptors such as the mannose-6-phosphate receptor [27]. The latter receptor also occurs in trophoblast cells [41]. Using megalin-expressing BN16 cells, we were now able to show that the megalin–V-ATPase pathway also underlies AGT uptake. Moreover, we observed that the PPI esomeprazole, such as the V-ATPase inhibitor bafilomycin A1, inhibits megalin-mediated internalization. Given that megalin-mediated AGT uptake underlies renal angiotensin generation [17], we speculate that a similar phenomenon may occur in the placenta. Here, we emphasize that the greater sensitivity of pre-eclamptic women to Ang II [11,42] may imply that placental Ang II levels do not necessarily have to be higher than those in healthy placentas in order to exert the same or even a stronger effect. Onda et al. showed that PPIs lower sFlt-1 synthesis in placental explants (confirmed in the present study), but were unable to link this to a direct consequence of V-ATPase inhibition with bafilomycin A1 [25]. Our data now reveal a new option: PPIs might suppress placental RAS activity by interfering either with AGT uptake, with (pro)renin transport, or both. Since lower placental Ang II levels are expected to result in diminished sFlt-1 production, this concept offers a novel explanation for the observation that PPIs lower circulating sFlt-1 in pre-eclamptic women (Figure 6).

## 4. Materials and Methods

### 4.1. Tissue Collection

The experiments were conducted using human placental tissue from women with a healthy, term pregnancy or with early-onset pre-eclampsia (<34 wk of GA) based on the ISSHP 2018 criteria [26]. All but one woman with early-onset pre-eclampsia delivered by caesarean section. The indication for caesarean section in healthy women was elective, due to either a previous caesarean section or breech position. In addition, all women gave informed consent prior to delivery. Placental tissue was collected immediately after delivery at the Erasmus Medical Center, Rotterdam, The Netherlands. Tissue sections were cut from the decidual side of the placenta and included decidua basalis and chorionic villi. They were snap frozen in liquid nitrogen and stored at −80 °C. Areas displaying necrosis, tissue damage, calcification, hematoma or tears were avoided. The study was exempted from approval by the local institutional Medical Ethics Committee according to the Dutch Medical Research with Human Subjects Law (MEC-2016-418 and MEC-2017-418).

### 4.2. Placenta Perfusion Experiments

The perfusion method used in this study was previously described by Hitzerd et al. [43]. In short, placentas were perfused at 37 °C with aerated (95% O_2_ and 5% CO_2_) perfusion medium consisting of Krebs–Henseleit buffer, supplemented with heparin (2500 IU/L; LEO Pharma B.V., Amsterdam, The Netherlands). The fetal circulation (closed-circuit; flow rate 6 mL/min) was established by cannulating the chorionic artery and corresponding vein of an intact cotyledon. Maternal circulation (closed-circuit; flow rate 12 mL/min) was created by placing four blunt cannulas in the intervillous space. Perfusion experiments were conducted in healthy and pre-eclamptic placentas. The healthy group consisted of placentas from uncomplicated, normotensive pregnancies in which the endothelin receptor antagonist ambrisentan (10 mg/L; Sigma-Aldrich, Schnelldorf, Germany) or the phosphodiesterase-5 inhibitor sildenafil (500 ng/mL; Pfizer Europe MA EEIG, Brussel, Belgium) was added to the maternal circulation at t = 0 [43,44]. The pre-eclamptic group consisted of placentas from early-onset pre-eclamptic pregnancies (<34 weeks) which were perfused with sildenafil or no drug. No drug-related differences in RAS component release were noted and, thus, all samples per group were combined. Samples from the maternal circulations were taken every 30 min (until 180 min) and immediately stored at −80 °C. To control the quality of perfusion, 100 mg/L of antipyrine (Sigma-Aldrich, St. Louis, MO, USA) and 36 mg/L of FITC-dextran (40 kDa; Sigma-Aldrich) were added to the fetal and maternal buffer, respectively. An experiment was considered successful when the fetal-to-maternal (F/M) ratio of antipyrine was >0.75 and the maternal-to-fetal (M/F) ratio of FITC-dextran was <0.03 at t = 180 min. The release of angiotensinogen, renin or prorenin was expressed per hour, correcting for the level that was present in the previous sample.

### 4.3. Placental Villous Explants

Freshly obtained placental tissue slices were cut from three different areas of each placenta and washed three times in cold phosphate-buffered saline (PBS; Lonza, Walkersville, MD, USA), after which the decidua and chorionic plate were removed. Tissue containing chorionic villi was then cut in explant blocks of 2 × 2 mm. Explants from the three different areas were combined in one well in 2 mL DMEM/F12 medium (Gibco, Thermo Fisher Scientific, Paisley, UK) containing 10% FCS (GE Healthcare, Eindhoven, The Netherlands), 1.95 g/L NaHCO_3_ and 100 μg/mL Primocin (Invivogen, San Diego, CA, USA) in 12-well plates, and equilibrated at 37 °C for 3 h at 8% O_2_ and 5% CO_2_. Thereafter, explants were transferred to a new plate and incubated with or without 100 μmol/L esomeprazole (Sigma-Aldrich) in the above medium for 24 h. sFlt-1 was measured in the medium using the human VEGFR1/Flt-1 DuoSet ELISA (R&D Systems, Minneapolis, MN, USA).

### 4.4. Placental Lysates

About 100 mg of placental tissue was homogenized with grind beads in 1 mL ice-cold buffer C (12 mmol/L NaH_2_PO_4_∙2H_2_O, 86.7 mmol/L Na_2_HPO_4_, 15.9 mmol/L NaCl) and 1x complete protease inhibitors (Roche, Mannheim, Germany) at 4 °C with a TissueLyser 24 (Shanghai Jingxin, Shanghai, China) 3 × 60 s at 60 Hz. The lysates were centrifuged at 14,000× *g* for 10 min at 4 °C. Then, the supernatant was transferred to a new tube and kept on ice. The pellet was resuspended in 200 μL of buffer C with protease inhibitors, and was additionally homogenized for 2 × 60 s at 60 Hz. After centrifuging at 14,000× *g* for 10 min at 4 °C, the second supernatant was added to the first supernatant, and the combined supernatants were stored at −80 °C.

### 4.5. Studies in Brown Norway Rat Yolk Sac Epithelial Cells

Brown Norway rat yolk sac epithelial cells (BN16) were cultured in Minimum Essential Media (MEM) (Gibco) supplemented with 1 × GlutaMAX (Gibco) and 10% FCS at 37 °C in a humidified incubator with 5% CO_2_. BN16 cells were seeded at a density of 2 × 10^5^ cells per well in 24-well plates and cultured for 48 h before carrying out experiments. To study the effect of proton pump inhibition on renin binding and internalization, BN16 cells were first washed twice with PBS, and then pre-incubated with MEM without FCS for 30 min at 37 °C. Next, cells were washed with ice-cold PBS twice, and incubated with 300 μL MEM containing 0.2 µg/mL of recombinant human renin (a gift from Actelion Pharmaceuticals, Allschwil, Switzerland) in the presence or absence of increasing concentrations of the H^+^/K^+^ ATPase inhibitor esomeprazole (Sigma-Aldrich) or the V-ATPase inhibitor bafilomycin A1 (Sigma-Aldrich) at either 4 °C (to quantify renin binding) or 37 °C (to quantify renin internalization). After 2 h of incubation, the culture medium was discarded, and the cells were washed twice with ice-cold PBS containing 0.5% BSA (Sigma-Aldrich) and twice with ice-cold PBS. Then, the cells were lysed with ice-cold PBS containing 0.2% Triton X-100 (SeeVa, Heidelberg, Germany) and 1× complete protease inhibitors. Lysate was centrifuged at 14,000× *g* for 10 min at 4 °C to remove any cell debris and the supernatant was stored at −80 °C until use.

To study whether megalin regulates angiotensinogen uptake, BN16 cells were transfected with 50 μmol/L of negative control (siNC) (Invitrogen, Paisley, UK) or siRNA against megalin (siMegalin) (Invitrogen) for 48 h by using RNAi max transfection reagent (Invitrogen). Then the cells were incubated with 10 μg/mL of His-tagged recombinant rabbit angiotensinogen (Sino Biological, Hong Kong, China) for 2 h. After incubation, cells were washed once with ice-cold PBS with 0.5% BSA and twice with ice-cold PBS, and then collected for immunoblotting.

### 4.6. Measurement of (Pro)renin and Angiotensinogen

Renin was measured in the placental - and BN16 cell lysates with the Renin III (Cisbio, Gif-sur-Yvette, France) immunoradiometric assay (detection limit 2 pg/mL). Total renin was measured in the lysates with the same assay, after activating prorenin with 10 μmol/L aliskiren (allowing its detection in the assay) at 4 °C for 48 h [45]. Prorenin in the lysates was calculated by subtracting renin from total renin. Given their low levels, renin and prorenin in the placental perfusates (the latter after its conversion to renin by trypsin) were measured by the more sensitive enzyme-kinetic assay as described before (detection limit 0.05 ng angiotensin I per mL per hour) [46]. Data were converted to pg/mL given that 1 ng Ang I/mL per hour equals 2.6 pg/mL renin [47]. AGT in the perfusates was measured as the maximum quantity of Ang I that was generated during incubation with excess recombinant renin (detection limit 0.5 pmol/mL) [46].

### 4.7. Immunoblotting

BN16 cells or placental tissue pieces were homogenized in RIPA buffer (150 mmol/L NaCl, 1% Triton X-100, 0.5% sodium deoxycholate, 0.1% SDS, 50 mmol/L Tris, pH 8.0) containing 1 × complete protease inhibitors with a TissueLyser (Polytron PT2100, Littau-Lucerne, Switzerland). Lysates were cleared by centrifugation at 14,000× *g* for 10 min at 4 °C. The total protein concentration in supernatant was determined by BCA assay (Pierce, Waltham, MA, USA). Equal amounts of protein (30–40 μg) were loaded and separated on Precast Midi Protein Gel (Bio-Rad, Hercules, CA, USA), and transferred to PVDF membranes using semi-dry Trans-Blot Turbo Transfer system (Bio-Rad). The blots were then probed with antibodies against angiotensinogen (1:100, Abbiotec, Shenzhen, China), GAPDH (1:5000, GeneTex, Hsinchu, China), β-actin (1:5000, Merck Millipore, Darmstadt, Germany), or megalin (1:500, Biotech, Wuhan, China), and detected by using Clarity Western ECL Substrate (Bio-Rad). The intensities of bands were analyzed using ImageJ.

### 4.8. RNA Isolation and qPCR Analysis

Total RNA was extracted using the Direct-zol RNA kit (Zymo Research, Irvine, USA). One microgram total RNA was reverse transcribed using QuantiTect^®^ Reverse Transcription Kit (Qiagen, Hilden, Germany). SYBR Green real-time quantitative PCR assays were performed on QuantStudio 7 Flex Real-Time PCR Systems (Thermo Fisher, Waltham, USA) using SYBR^®^Premix Ex TaqTM II kit (Qiagen, Venlo, The Netherlands). Primers used in the study are *(P)RR* (forward: 5′-TCTCAGTTCACTCCCCCTCAA-3′; reverse: 5′-GATGCTTATGACGAGACAGCAAG-3′), *renin* (forward: 5′-GCCGTCTCTACACTGCC-TGT-3′; reverse: 5′-GGAGGGTGAGTTCTGTTCCA-3′), *AGT* (forward: 5′-TCAACACCT ACGTCCACTTCC-3′; reverse: 5′-CACTGAGGTGCTGTTGTCCA-3′), *AGT* primer 2 (forward: 5′-ACCTACGTCCACTTCCAAGG-3′; reverse: 5′-GTTGTCCACCCAGAACTCCT-3′), *AGT* primer 3 (forward: 5′-ACAAGGTGGAGGGTCTCACT-3′; reverse: 5′-TGGATGGTCCGGGGAGATAG-3′), *ACE* (forward: 5′-CCAACCTCGATGTCACCAGT-3′, reverse: 5′-TCGACCCTTCCCAGAACTC-3′), *AT1R* (forward: 5′-TGACAGTCCAAAGGCTCCA-3′, reverse: 5′-TTTGATCACCTGGGTCGAAT-3′), *AT2R* (forward: 5′-GGCAACTCCACCCTTGCCACT-3′, reverse: 5′-TGCCAGAGATGTTCACAAGCCCGA-3′), *ACE2* (forward: 5′-TTCTGTCACCCGATTTTCAA-3′; reverse: 5′-TCCCAACAATCGTGAGTGC-3′), *megalin* (forward: 5′-CTGCTCCTGGCTCTCGTC-3′; reverse: 5′-CTTTGGTCCCATCACACCTC-3′), *β-actin* (forward: 5′-CTGCTCCTGGCTCTCGTC-3′; reverse: 5′-CTTTGGTCCCATCACACCTC-3′), Pre-mRNA Processing Factor 38A (*PRPF38A*) (forward: 5′-GTTAAGGTTTGTGGGTGGCG-3′; reverse: 5′-AGCATGCGGACATACTTGAAATC-3′), DExD-Box Helicase 50 (*DDX50*) (forward: 5′-GCCTCCTGAAAGGAAATATGG-3′; reverse: 5′-AGTATCCAGTCGGAATCATGC-3′), *Flt-1* (forward: 5′-ACAATCAGAGGTGAGCACTGCAA-3′; reverser: 5′-TCCGAGCCTGAAAGTTAGCAA-3′) (Invitrogen). *β-actin, PRPF38A* and *DDX50* were used as housekeeping genes for calculating the relative expressions of target genes, and the geometric mean of the three relative expressions were used for calculating the arbitrary unit (A.U.). 

### 4.9. Statistics

Data are provided as mean ± SEM or geometric mean and range. When comparing differences between two groups, Student’s *t*-test was used. One-way ANOVA or two-way ANOVA with Bonferroni post-modification was used when comparing differences between more than two groups. The Kolmogorov–Smirnov test was used to verify normal distribution. For data that were non-normally distributed, a non-parametric *t*-test (Mann–Whitney U test) was used to compare the differences between groups. Categorical variables were evaluated by Chi-square test. *p* < 0.05 was considered significant. Data below detection limit were assumed to equal the detection limit.

## Figures and Tables

**Table 1:** Clinical characteristics of placentas.

Characteristic	Healthy (*n* = 25)	EoPE (*n* = 17)
Maternal age (years)	31 (28–36)	30 (26–32)
Nulliparity/multiparity (n)	6/19	11/6 *
Ethnicity (n = Caucasian/other)	15/10	12/5
Highest diastolic blood pressure (mmHg)	80 (74–82)	109 (100–116) *
Urinary protein-to-creatinine ratio	ND	351 (92–861) *
Mode of delivery (n = caesarean/spontaneous)	25/0	16/1
Gestational age (weeks)	39.0 (38.7–39.1)	29.9 (28.8–31.8) *
Sex (n = female/male)	7/18	9/8
Birth weight (g)	3465 (3175–3763)	1125 (920–1300) *
Birth weight centile < 10th (n)	0	9
Placental weight (g)	623 (568–729)	295 (232–347) *

Data are shown as n (number of cases) or median (interquartile range). * *p* < 0.05. ND, not detected; EoPE, early-onset pre-eclampsia.

## Data Availability

All data supporting the reported results can be acquired from the corresponding author upon reasonable request.
